# Elongation factor 2 in cancer: a promising therapeutic target in protein translation

**DOI:** 10.1186/s11658-024-00674-7

**Published:** 2024-12-20

**Authors:** Xuechao Jia, Chuntian Huang, Fangfang Liu, Zigang Dong, Kangdong Liu

**Affiliations:** 1 Henan International Joint Laboratory of TCM Syndrome and Prescription in Signaling, Traditional Chinese Medicine (Zhong Jing) School, Henan University of Chinese Medicine, Zhengzhou, 450046 Henan China; 2 Department of Pathophysiology, School of Basic Medical Sciences, Zhengzhou University, Zhengzhou, 450000 Henan China; 3 Department of Pathology and Pathophysiology, School of Medicine, Henan University of Chinese Medicine, Zhengzhou, 450046 Henan China; 4 China-US (Henan) Hormel Cancer Institute, Zhengzhou, 450000 Henan China; 5 Department of Medical Genetics and Cell Biology, School of Basic Medical Sciences, Zhengzhou University, Zhengzhou, 450000 China; 6 Tianjian Laboratory of Advanced Biomedical Sciences, Zhengzhou, 450001 Henan China; 7 The Collaborative Innovation Center of Henan Province for Cancer Chemoprevention, Zhengzhou, 450000 Henan China

**Keywords:** Elongation factor 2, Cancer, Inhibitors, Protein elongation, Regulators

## Abstract

Aberrant elongation of proteins can lead to the activation of oncogenic signaling pathways, resulting in the dysregulation of oncogenic signaling pathways. Eukaryotic elongation factor 2 (eEF2) is an essential regulator of protein synthesis that precisely elongates nascent peptides in the protein elongation process. Although studies have linked aberrant eEF2 expression to various cancers, research has primarily focused on its structure, highlighting a need for deeper exploration into its molecular functions. In this review, recent advancements in the structure, guanosine triphosphatase (GTPase) activity, posttranslational modifications, regulatory factors, and inhibitors of eEF2 are summarized. These findings provide a comprehensive cognition on the critical role of eEF2 and its potential as a therapeutic target in cancer. Furthermore, this review highlights important unanswered questions that warrant investigation in future research.

**Graphical Abstract:**

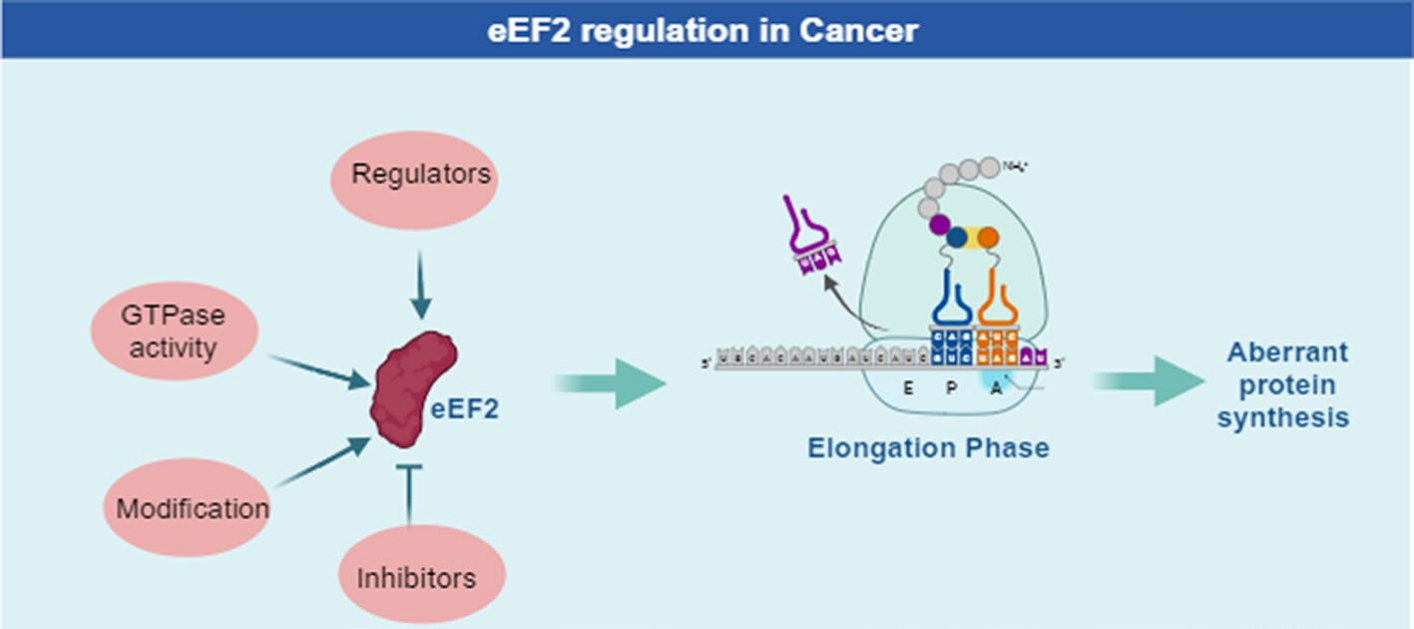

**Supplementary Information:**

The online version contains supplementary material available at 10.1186/s11658-024-00674-7.

## Introduction

Translation elongation is tightly regulated by a complex network of factors, including ribosomal proteins, transfer RNA (tRNA) synthetases, and other factors. Dysregulation of these factors impacts protein translation and contributes to cancer development and progression [1]. The aberrant translated proteins subsequently induce the continuous activation of oncogenic signaling in cancer, leading to the dysregulating of both the oncogenic genes and tumor suppressor genes. This vicious cycle of regulation usually promotes cancer progression, metastasis, and drug resistance. In the past, the roles of aberrantly translated genes have been extensively studied, whereas the significance of proteins involved in the translational process has often been underestimated.

Eukaryotic elongation factor 2 (eEF2) is an essential elongation regulator that participated in protein translation process [2]. It belongs to a member of the highly conserved guanosine triphosphatase (GTPase) family, which is responsible for the precise reading of messenger RNA (mRNA) frame [3, 4]. Under normal conditions, eEF2 binds mRNA and facilitates elongation through interactions with GTP and other essential factors [5]. Elongation process is activated upon eEF2 binding with GTP and is inactivated when the eEF2-GDP form dissociates from the ribosome, thereby, ensuring the precise enrollment of elongation process [6]. During this process, hydrolysis of GTP by eEF2 leads to a conformational change and it is perceived to play an important role in the regulation of elongation rate [7]. Phosphorylation at Thr56 by eEF2k inhibits eEF2, suppressing the elongation process [8]. Recent studies have shown that eEF2 is overexpressed in several cancers, including gastrointestinal cancers and lung adenocarcinoma, where elevated levels correlate with increased tumor incidence and poorer patient prognosis [9, 10]. This underscores the clinical relevance of eEF2 as a therapeutic target in cancer. Supporting this, a phase 1 clinical trial (NCT01061645) explored MOC31-PE, an immunotoxin designed to target EpCAM-positive carcinomas by inhibiting protein synthesis through eEF2 modification [11]. By disrupting protein synthesis in tumor cells, this approach demonstrated potential therapeutic benefits in advanced carcinomas, illustrating the crucial role eEF2 plays in cancer progression. These findings emphasize the need for further research into eEF2-targeting drugs, reinforcing its potential as a target in cancer treatment.

Despite the significance of eEF2 in cancer, its precise functions and underlying mechanisms remain elusive, highlighting the need for further investigation. Therefore, elucidating the role of eEF2 and discovering its inhibitors in cancer is poised to greatly benefit the clinical outcomes of patients with cancer in the future. While numerous reviews have focused on the molecular structures of eEF2, limited attention has been given to its other functions. Here, we provided a comprehensive overview encompassing eEF2’s protein structure, functions, GTPase activity, various modifications, regulators, and inhibitors. Furthermore, we raised valuable insight into the current understanding of eEF2 while emphasizing its potential as a target for developing novel therapies against cancer.

## The roles of eEF2 in cancer transformation and progression

eEF2 is pivotal in cancer transformation and progression, influencing protein synthesis dynamics across various tumor types. In ARID1A-deficient tumors, such as bladder cancer, the loss of ARID1A leads to a transcriptional-translational conflict, resulting in an accumulation of pro-proliferative transcripts while inhibiting eEF2 function. Enhancing eEF2-mediated translation resolves this conflict, promoting the synthesis of pro-proliferative mRNAs and driving cancer progression, thereby highlighting eEF2 as a potential therapeutic target [12]. Similarly, in colorectal cancer, mutations, such as Rpl24^Bst^, increase eEF2 phosphorylation, suppressing protein synthesis and tumor growth, which emphasizes eEF2’s role in cancer cell proliferation [13]. eEF2 also contributes to chemotherapy resistance, as seen in oxaliplatin-resistant colorectal cancer [14]. Here, endoplasmic reticulum stress leads to eEF2 modifications that activate the PERK pathway, inducing senescence and drug resistance. Targeting these modifications with inositol hexaphosphate (IP6) has shown promise in reversing drug resistance. In chronic lymphocytic leukemia (CLL), inhibiting mammalian target of rapamycin complex 1 (mTORC1) reduces eEF2 activity, leading to decreased synthesis of cell cycle proteins and halting CLL progression [15].

Moreover, in hepatocellular carcinoma (HCC), eEF2 enhances translation of HMGB2 mRNA, promoting tumor growth and metastasis. Targeting this mechanism with Panobinostat has been effective in inhibiting HCC progression [16]. Additionally, eEF2 facilitates the translation of ribosomal proteins critical for p53-mediated apoptosis during metabolic stress, reinforcing tumor survival mechanisms [17]. The interaction of eEF2 with PRMT7 also drives invasion and metastasis in non-small cell lung cancer (NSCLC) [18]. Thus, eEF2 emerges as a crucial mediator in the complex signaling pathways underlying cancer progression and treatment resistance.

## Structure and function of eEF2

**Fig. 1:**
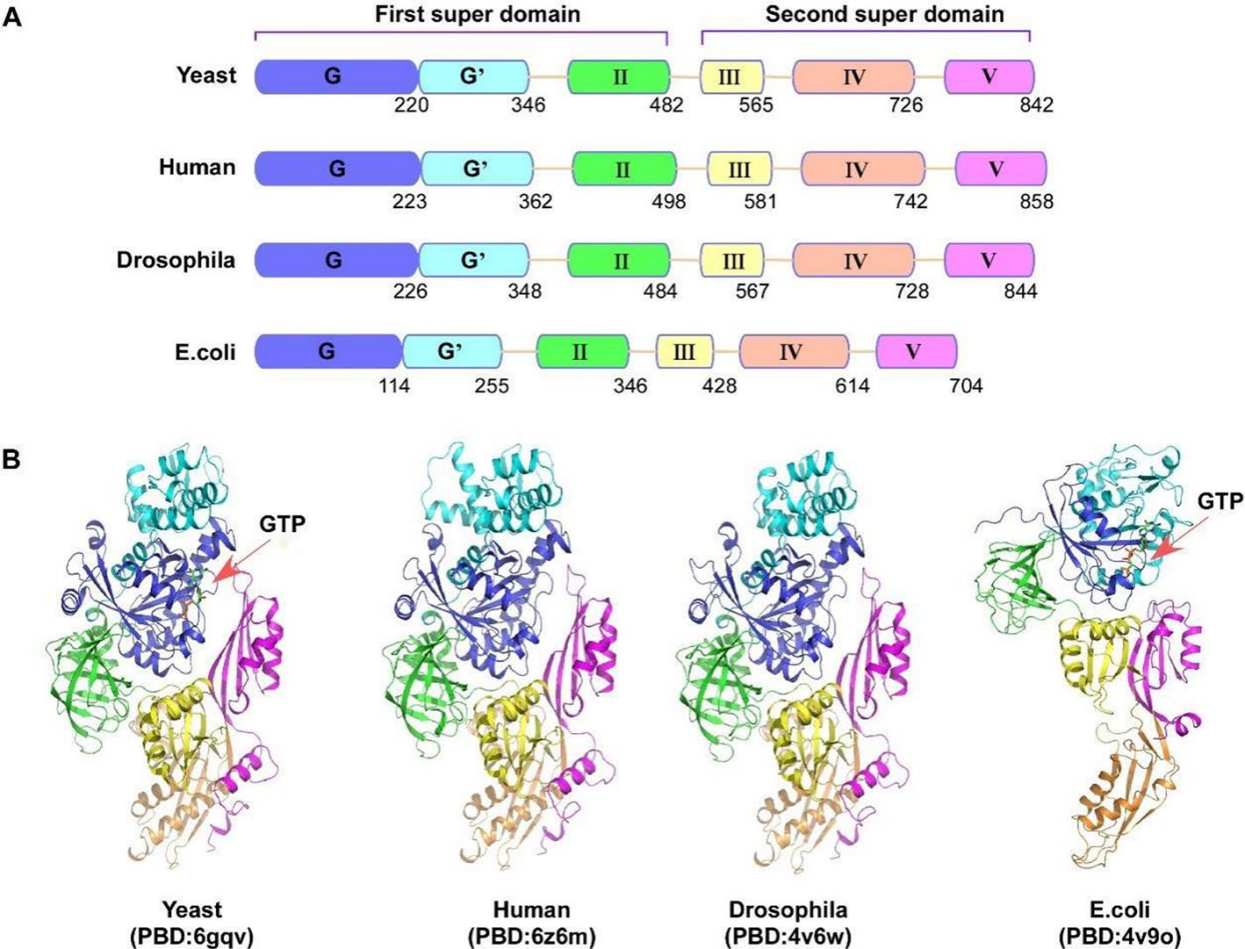
**A** The protein structures similarity of eEF2 between yeast, human, *Drosophila*, and *E*. *coli*. **B** The amino composition and domain similarity: domain I (G, G′); domain II, domain III, domain IV, and domain V. The three-dimensional (3D) structure of eEF2 and the binding site of GTP in the structure. The colors of different protein domains are consistent in **A** and **B**. The figure was created with ACDSee Systems Canvas

During the elongation process, eEF2 undergoes orderly dynamic changes in its structure, regulated by various translation components [5]. Although extensive research has been conducted on the structure of eEF2, its functions in cancer have received less attention. The schematic diagram (Fig. 1A, Supplementary 1) illustrates the protein structures and similarities of eEF2 between yeast, human, *Drosophila*, and *Escherichia coli*. As depicted in the schematic diagram, eEF2 consists of five intricate domains that can be classified into two loosely associated super domains [19]. These domains include a GTPase domain I (GG′) at the N-terminal, a rigid block form of domain II–V at the C-terminal region, which undergoes conformational rearrangements during the protein elongation cycle [3]. The GG′ II domains belong to the first super-domain while the III, IV, and V domains belong to the second super-domain. The first super-domain is crucial for maintaining the stability of C-terminal super-domains III–V during folding and isolation processes of eEF2 [20]. Furthermore, crystal structure comparisons reveal gross conformational changes in eEF2 upon binding to ribosomes. Within each super-domain grouping, the second super-domain is observed to exhibit a hinge-like motion, which induces a ratchet-like rotation of the ribosome’s small subunit [21].

During the translation process, evidence suggests that different subunits, ribosomal RNA (rRNA) helix or ribosomal proteins interact with specific domains of eEF2 [22]. It is reported that GTP-binding motif resides within GG′ domains of eEF2 (Fig. 1B). With higher resolution cryo-electron microscopy (cryo-EM), the GG′ domain of human and *Drosophila* eEF2 has been observed to interact with the amino-terminal extension of ribosomal stalk proteins at L10 and L11 [5]. Although the overall conformation and interaction of human and *Drosophila* eEF2 on the ribosome are similar to those for yeast, lower resolution yeast eEF2-80S complexes do not reveal the detailed interaction sites [23]. In contrast, a similar interaction between the C terminus stalk proteins L7 and L12 of the 60S ribosomal subunit and the GG′ domain of EF-G is observed in bacteria [24, 25]. During translation, GTP hydrolysis by the GG′ domain promotes ribosome movement along mRNA [26]. The intricate network formed by domain II and III of eEF2 with helix 5 of 18S rRNA during the small subunit (SSU) body rotation is crucial. Domain III attaches to uS12 protein located on the SSU shoulder and is pulled by the SSU body back-rotation, thereby switching to domain II in the G-domain [27]. Additionally, domain IV protrudes deeper into A-site of SSU during late-translocation process [28]. The N terminus of the eukaryote-specific protein eS30 and the decoding protein uS12 interact with domain IV of eEF2 to provide supplementary stabilization and enhance the decoding site conformational changes [29]. Compared with early translocation-intermediate complex, domains I and V of eEF2 are sturdily anchored on large subunit (LSU) rRNA at late translocation-intermediate complex stage [5].

Despite extensive studies on its structure, the understanding of human eEF2 remains incomplete. Accurately determining the conformational changes in eEF2 during protein translation process is vital for comprehending its detailed functions. Moreover, screening inhibitors on the basis of protein crystal structure has emerged as an effective strategy for cancer-targeting therapy research in recent years, thus targeting different domains of eEF2 to screen inhibitors could be a novel approach to impede cancer growth.

## The GTPase activity of eEF2

eEF2, a member of the GTPase superfamily, is activated upon binding to GTP during the translocation phase and disassociates from the ribosome in its inactive form(eEF2-GDP) [6]. The complex formation between eEF2 and GTP stabilizes the hybrid state by inducing conformational changes in eEF2 and promotes rapid hydrolysis of GTP [30]. Despite evidence showing that inhibiting eEF2’s GTPase activity can suppress protein translation, targeted inhibition of eEF2’s GTPase activity in cancer has not been given due attention in previous studies.

Research on the function of eEF2’s GTPase activity has evolved through various twists and turns, primarily relying on studies involving eEF2 and EF-G. In the 1970s, it was proposed that eEF2 acts similarly to a regulatory G protein as a GTPase protein [31]. Translocation was believed to occur spontaneously but at a slow rate unless catalyzed by eEF2’s GTPase activity [32]. However, these views are challenged by evidence suggesting that tRNA translocation is preceded by GTP hydrolysis coupled with phosphate release [33]. It has been suggested that binding of eEF2 without subsequent GTP hydrolysis plays an important role in catalyzing tRNA translocation and promoting conformational changes in eEF2 [34]. Subsequently, it was demonstrated that binding of eEF2 to ribosomes occurs prior to GTP hydrolysis and sufficient for promoting or stabilizing the ribosomal conformation [35, 36]. Later studies reported that GTP hydrolysis induces movement of domain IV within eEF2 resulting in decoupling mRNA–tRNA complexes from decoding centers allowing transnational movements via head rotation of small rRNA subunits [21]. Previous studies have also employed antibiotics, non-hydrolysable GTP analogs or mutated EF-G to investigate the effect of GTP hydrolytic on the ribosome and found that the translocation speed is accelerated in those conditions [37, 38]. Nevertheless, there are reports indicating that the translocation rate is significantly higher in the presence of EF-G and GTP compared with mere GTP analogs or inactive EF-G [39]. Whether the GTPase activity of eEF2 plays an important role in the elongation process has aroused controversy. Carbone et al. concluded that the GTP-catalyzed translocation structures were either lowly-populated or not populated in previous mechanistic models, and they subjected these findings to two different models [40]. In the first model, they suggested that GTP hydrolysis induces a large-scale conformational change leading to tRNA movement [26, 41, 42]. The second model proposed that EF-G acted like a pawl which rectified the motions of the ribosome [43]. They demonstrated the importance of GTP hydrolysis in dissociating eEF2/EF-G from the ribosome but did not uncover the detailed functional phase of eEF2. Excitingly, recent crystal structure studies by Djumagulov and Kišonaitė et al. suggest that GTP hydrolysis occurs at the late stages of translocation [5, 44]. Overall, translation elongation requires eEF2/EF-G and GTP hydrolysis; thus, molecules or inhibitors affecting their activity may lead to translocation defects and loss of cell viability. However, further investigation is needed to fully understand the dynamic functions of eEF2 in translation processes. Additionally, inhibiting eEF2’s GTPase activity of could be a potential therapeutic strategy for patients with highly expressed eEF2 in cancer.

## The posttranslational modification of eEF2

**Fig. 2:**
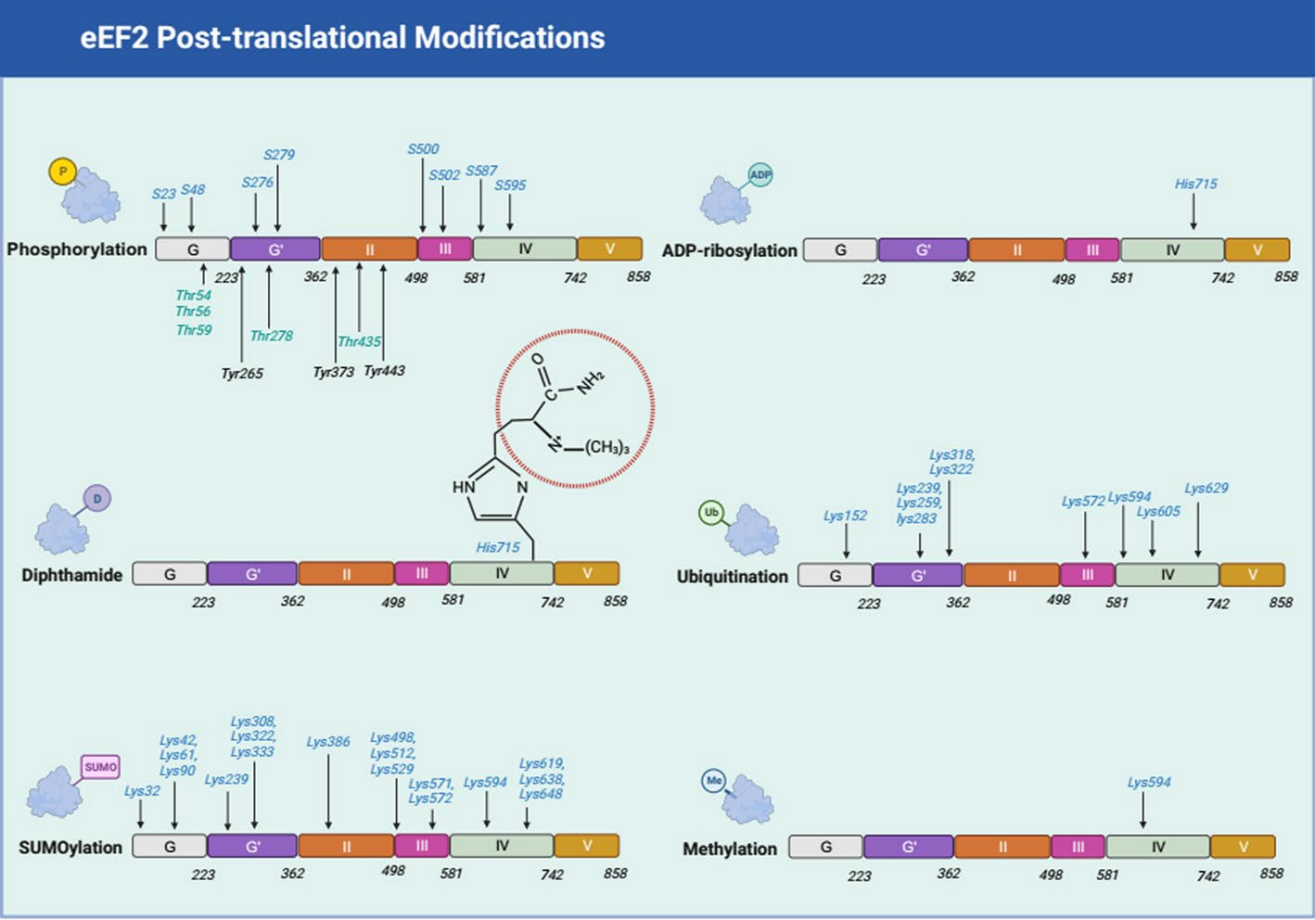
The common modification categories and modification amino sites of eEF2. The detail amino sites were shown beside the corresponding modification. The figure was created with BioRender.com

Different modifications and the associated molecular signaling pathways of eEF2 have been found to be aberrant in various types of cancers (Fig. 2). In the qPhos protein dynamic modification database, eEF2 has been identified as being subjected to diverse modifications in different cancer cells, including phosphorylation, diphthamide modification, SUMOylation, and ubiquitylation, among others [45]. We have categorized these modifications on the basis of their significantly altered values and specific sites of modification (Supplementary 2). This supplementary highlights that phosphorylation is the most extensively investigated modification of eEF2, whereas other modifications have only been observed in a limited number of studies. Additionally, we present a comprehensive overview of research studies on eEF2 posttranslational modifications, including phosphorylation, diphthamide modification, SUMOylation, ADP-ribosylation, and methylation (Table 1).

**Table 1:** The association between different eEF2’s PTM modification and disease/physiological dysfunction

PTM modification	Physiological dysfunction	Disease	Refs.
Phosphorylation	Facilitates T56 phosphorylation by recruiting eEF2K to eEF2	Cervical cancer	[46]
	Triggering T56-phosphorylation	Breast cancer	[47, 48]
	Inhibited expression of MCL1, cyclin A, and cyclin D2	Chronic lymphocytic leukemia	[26]
	T56 phosphorylation, reducing protein synthesis by 40%	Colorectal cancer	[24]
	Expression of tumor cell regulators, EGF receptors, cyclin A, and MMP-9	Melanoma	[48]
Diphthamide modification	DPH5-related diphthamide-deficiency	Embryonic lethality	[49]
	Regulation of ribosome biogenesis via activating Ras pathway	Gut tumor	[50]
	Diphthamide deficiency impairing tagraxofusp’s ability	Hematologic and myeloid malignancies	[51, 52]
SUMOylation	Maintaining protein stability and positively	Lung adenocarcinoma	[9]
	Enhanced the protein cleavage and nuclear translocation of eEF2	Cervical cancer	[53]
	Translocated eEF2 into cardiomyocytes nucleus	Myocardial ischemia reperfusion	[54]
	Maintaining eEF2 in a dephosphorylated state	Cardiomyocytes	[55]
ADP-ribosylation	MAPK and PKC signaling pathways, inhibiting protein synthesis	Liver cancer	[56]
	Resistance to PE and DT	Breast cancer	[57]
	Blocked ADP-ribosylation, tumor resistance	Myeloid malignancies	[58]
Methylation	Methylation of eEF2 at lysine 525, promote protein synthesis	Lung adenocarcinoma	[59]

### The phosphorylation modification of eEF2

The phosphorylation of eEF2 has been observed to be dysregulated in various cancers, where the phosphorylated status of eEF2 represents an inactive form that hinders its binding to ribosomes and affecting translation and poly(U)-directed polyphenylalanine synthesis [12, 60]. Conversely, dephosphorylation of eEF2 enhances its internal activity and promotes peptide chain elongation during translation process. Rapid and significant dephosphorylation of eEF2 has been observed in cells exposed to insulin [61].

Thr56 is the most observed phosphorylation site of eEF2. The phosphorylation site of Thr56, located at GG′ domains of eEF2, is phosphorylated by eEF2k, thereby disrupting protein elongation [62]. Recent investigations by Hizli and Seidl have revealed another phosphorylation site of eEF2, Ser595 [46, 47]. They found that Ser595 phosphorylation was highly enriched in the mitosis phase in U2OS and HeLa cells. After being phosphorylated by CDK1 and CDK2, Thr56 was subsequently activated for phosphorylation. In chronic lymphocytic leukemia (CLL), targeting the eEF2/eEF2K axis through phosphorylation effectively suppresses disease progression by modulating proteins critical for cell proliferation [15]. Similarly, in colorectal cancer (CRC), increased eEF2 phosphorylation, as demonstrated in Rpl24^Bst^ mutant mice, significantly limits tumorigenesis, positioning translation elongation as a key therapeutic target [13]. Furthermore, in melanoma, β2-AR activation by (R, R')-MNF enhances eEF2 phosphorylation, leading to reduced expression of tumor regulators such as EGFR, cyclin A, and MMP-9, thereby suppressing tumor growth and progression [48]. Collectively, these findings underscore the central role of eEF2 phosphorylation in inhibiting protein synthesis and tumor growth across multiple cancer types, making it a promising target for therapeutic intervention.

Although phosphorylation modifications of eEF2 have been extensively reported, most studies focus solely on Thr56 and Ser595, despite the existence of many other phosphorylation sites in the qPhos database. However, the functions of these additional phosphorylation sites have not been evaluated. In inhibitor studies, 1-benzyl-3-cetyl-2-methylimidazolium iodide (NH125) has been identified as a potential inhibitor of eEF2k in previous report [63]. Nonetheless, another study discovered that NH125 inhibited the proliferation ability of various cancer cells and evoked the accumulation of phosphorylated eEF2 [64]. Thus, this study supposes that NH125 inhibits the cancer cells not via targeting eEF2k. Additionally, it has been observed that the Thr56 phosphorylation status of eEF2 exhibits an inverse pattern between the cells and in vitro kinase assays, indicating that other phosphorylation sites may be more suitable for kinase activity study. Therefore, it is imperative to uncover the functions of other phosphorylation sites of eEF2 in cancers.

### The diphthamide modification of eEF2

Diphthamide modification is a conserved and unique posttranslational modification in eEF2, where the modified amino site is located at histidine715 residue in human eEF2, His702 in *Drosophila*, and His699 in yeast. During translocation, this modification disrupts the affinity between the mRNA–tRNA duplex and decoding center [5].

Diphthamide is targeted by diphtheria toxin, exotoxin A, and cholix toxin [2, 65]. The diphthamide is ADP-ribosylated and subsequently inactivates the protein synthesis function of eEF2 upon treatment by the virulent toxins [66]. The diphthamide modification is reported to interact and stabilize the codon–anticodon complex during translation [5]. The process of diphthamide modification and synthesis includes four steps and is primarily regulated by the participation of diphthamide biosynthetic protein (Dph)1-Dph7 [67–71]. The diphthamide modification of eEF2 is reported to play an important role in eukaryotes and deleting the modification enzymes in mice contribute to severe developmental defects or embryonic lethality [49, 72, 73]. Ortiz et al. found that diphthamide modification defects in eEF2 increased the occurrence of frameshifting and attenuated the suppressive effect of toxins [74]. Pellegrino et al. utilized cryo-EM methods to demonstrate that the diphthamide post-modification was vital for maintaining accurate reads of mRNA reading frame through structural insight [75].

In recent years, studies have investigated the role of diphthamide modification in eEF2 in various processes, including cancer progression and translation regulation. For example, Kayoko et al. found that eEF2 diphthamide modification was involved in gut tumor progression in adult *Drosophila* and discovered the underlying mechanism that diphthamide modification regulated the ribosome biogenesis process by activating the Ras pathway [50]. Togami and Gondek et al. demonstrated that restoring diphthamide modification deficiency of eEF2 could enhance the therapeutic sensitivity of interleukin 3 receptor to tagraxofusp in hematologic and myeloid malignancies [51, 52]. Zhang Yugang et al. verified that under nutrient-rich conditions, diphthamide modification of eEF2 formed a positive feedback loop, thus promoting translation by targeting the rapamycin complex 1 (TORC1)/mammalian TORC1 (mTORC1) signaling pathway [76]. Above all, despite extensive investigation into the positive regulation of diphthamide modification, the protein responsible for negative regulation of diphthamide modification and ribosylation has yet to be identified.

### The SUMOylation of eEF2

Despite the increasing focus on other eEF2 protein modifications, research on the SUMOylation of eEF2 remains limited. To date, aside from its degradation intermediate of eEF2, the biological regulatory role of SUMOylation in eEF2 has seldom been reported. In 2011, Chen Chih-Yi et al. observed SUMOylation of eEF2 in lung adenocarcinoma cancer cells, which was crucial for protein stability and positively correlated with cisplatin resistance in lung adenocarcinoma [9]. Similarly, Yao Qi et al. reported in 2014 that SUMOylation of eEF2 enhances its cleavage and nuclear translocation of eEF2, ultimately inducing morphological changes in the nuclei of HeLa cells [53]. It was found that overexpression of SUMO proteins resulted in the accumulation of small fragments of eEF2, while knocking down Csk resulted in a decrease in eEF2 fragments. The amino sites Lys322 and Lys529 of eEF2 were identified as SUMOylation modification sites of Csk, indicating a crosstalk between eEF2 SUMOylation and phosphorylation. In 2017, Zhang chao et al. discovered that phosphorylated eEF2 could be SUMOylated and translocate into cardiomyocytes nucleus in myocardial ischemia reperfusion process [54]. Similarly, they also indicated that HSP70 suppressed eEF2 SUMOylation by maintaining eEF2 in a dephosphorylated state in cardiomyocytes [55]. However, the precise mechanism by which eEF2 SUMOylation, along with phosphorylation, regulates the cleavage and nuclear translocation remains unclear. Further exploration is needed to determine how HSP70 suppresses the SUMOylation of eEF2.

### The ADP-ribosylation of eEF2

ADP-ribosylation of eEF2 disrupts its normal function in protein synthesis by inhibiting phosphorylation at S595 and promoting phosphorylation at T56, leading to prolonged translation arrest [47]. This modification affects cellular growth and survival pathways, contributing to tumor progression, especially in response to stress or toxins. Toxins, such as Cholix, further illustrate this by ADP-ribosylating eEF2, inhibiting protein synthesis, and inducing apoptosis in HepG2 liver cancer cells through the MAPK and PKC signaling pathways, which elevate TNF-α levels [56]. In cancer biology, eEF2 modification plays a crucial role, as demonstrated by studies using diphthamide-deficient MCF7 breast cancer cells, where the loss of diphthamide blocked ADP-ribosylation, conferring resistance to Pseudomonas exotoxin A (PE) and diphtheria toxin (DT) [57]. Additionally, the fusion toxin tagraxofusp, which targets CD123 in tumor cells, exploits eEF2 ADP-ribosylation [58]. Resistance to this therapy can arise from DPH1 downregulation, but azacitidine restores DPH1 expression, re-sensitizing cells to treatment. These studies highlight the critical role of eEF2 ADP-ribosylation in both cancer progression and treatment strategies.

### The methylation of eEF2

Methylation of eEF2 at lysine 525 (eEF2K525me3), catalyzed by FAM86A, is crucial for mRNA translation and tumor progression in lung adenocarcinoma (LUAD) [59]. This modification enhances eEF2’s interaction with the ribosome, promoting protein synthesis and cancer cell proliferation. High levels of eEF2K525me3 and FAM86A overexpression correlate with advanced LUAD stages, suggesting that the FAM86A-eEF2K525me3 axis could be a promising therapeutic target. Structural studies, including X-ray crystallography and AlphaFold modeling, have highlighted the importance of the FAM86 domain in this methylation process. A specific antibody against eEF2K525me3 further supports the role of FAM86A in enhancing eEF2’s function [77]. Additionally, SMYD2, another lysine methyltransferase, also methylates eEF2, and its inhibition by BAY-598 reduces eEF2 methylation [78]. This underscores the broader significance of these methylation processes in regulating protein translation and cancer progression.

## Regulation factors of eEF2

**Fig. 3:**
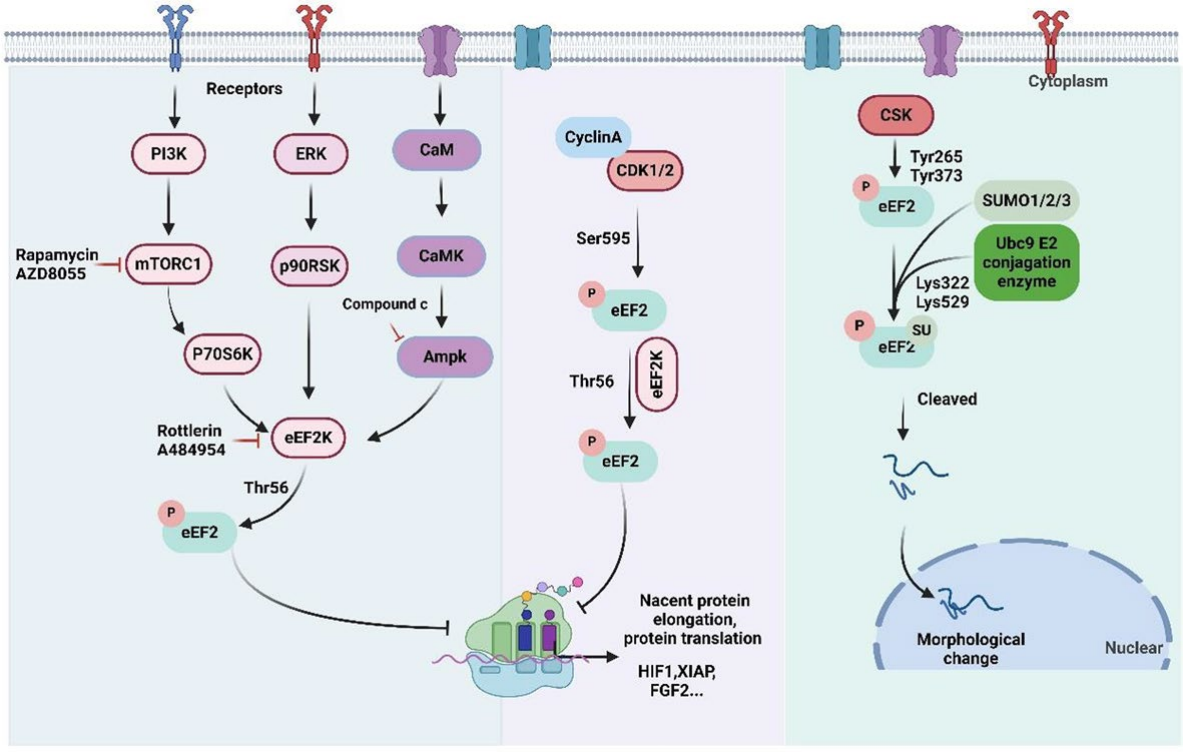
The directed kinases and detail amino sites that regulating eEF2 phosphorylation. eEF2K, CDK1/2 and CSK elevated the phosphorylation of eEF2. The figure was created with BioRender.com

The protein eEF2 is subject to regulation by multiple molecules, including eEF2k, CDK1/2, Csk, PP2A, and others. Abnormal regulation of these molecules has been found in various cancers, as they control the accurate elongation process in a coordinated manner. eEF2k, the most recognized kinase, phosphorylated eEF2 at Thr56, resulting in the down-regulation of protein translation and increasing the translation accuracy in mammals [79]. The inactive or active status of eEF2k alternates to ensure the elongation function of eEF2, which is modulated by various signal pathways and factors (Fig. 3). The classical rapamycin complex1 (TORC1) pathway, PI3K pathway, extracellular signal-regulated kinase (ERK)/ MAP kinase pathway and AMPK pathway has been reported to be involved in the activation regulation of eEF2k, thus affecting the precision translation [80–84]. These associated signals can be influenced by various external and internal factors. As illustrated in the Fig. 3, the internal or external factors, such as low pH, genotoxic stress, nutrient deprivation, 2-deoxyglucose, lopinavir, and hypoxia [85–89] evoke the activity of eEF2k and increase eEF2 phosphorylation, while ghrelin, insulin, serum, ceramide, essential amino acids (leucine), lithium, and high glucose reduce phosphorylation of eEF2 [90–95]. In addition to eEF2k, recent studies have identified other kinases that phosphorylate eEF2 at different phosphorylation sites. For instance, eEF2 is phosphorylated at serine 595 by CDK1 and CDK2, linking this phosphorylation to cell cycle regulation and translation control [46, 47]. In addition, eEF2 is also reported to be phosphorylated at Tyr-265 and Tyr-373 by Csk, a kinase phosphorylated Src family kinases, thus leading to eEF2 cleavage into small fragments and inducing nuclei aneuploidy change in HeLa cells [53].

**Fig. 4:**
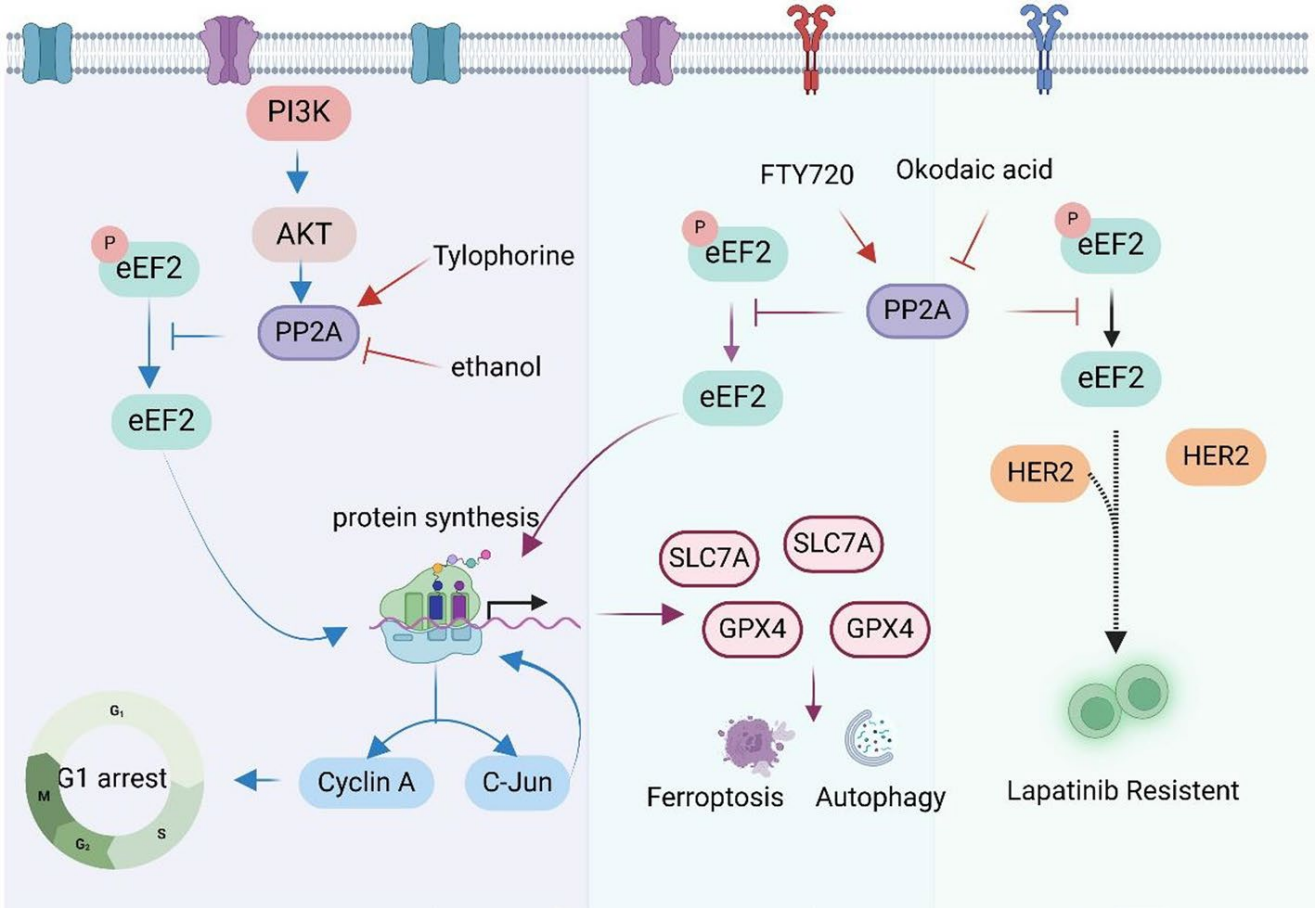
The signal pathways of PP2A regulates the dephosphorylation of eEF2. The figure was created with BioRender.com

Protein phosphatase 2A (PP2A) is a critical phosphatase that regulates the dephosphorylation of eEF2 in eukaryotic cells [96, 97]. Multiple studies have demonstrated the involvement of PP2A in eEF2 dephosphorylation and its impact on various cellular processes (Fig. 4). For instance, in multiple myeloma cells, fingolimod (FTY720), a structural modification product of myriocin, was reported to activate PP2A via suppressing the Tyr307 amino phosphorylation of PP2A [97]. Subsequently, the phosphorylated level of eEF2 was observed to decrease in the carcinoma cells leading to reduced protein synthesis levels of SLC7A11 and GPX4, and consequently, inducing ferroptosis and autophagy [97]. In cardiac myocytes, PP2A is found to function along with angiotensin II, thereby stimulating the dephosphorylation and activation eEF2 through PI3K and MAPK pathways [98]. In HONE-1, NUGC-3 and HepG2 carcinoma cells, tylophorine, which is extracted from Tylophora indica, has been reported to increase the dephosphorylated protein level of eEF2, thereby accumulating the protein level of c-jun and cyclin A, thus arresting the cell cycle at G1 phase [99]. Finally, they concluded that tylophorine arrested the carcinoma cell cycle through PI3K, PDK1, PP2A, eEF2, and c-jun signaling pathway. An evaluation of the mechanisms of ethanol on protein synthesis in cells confirmed that ethanol increased eEF2 phosphorylation via suppressing the activity of PP2A independent of eEF2K pathway [100]. Additionally, the protein activity of PP2A was observed to elevate in HER2-positive and trastuzumab- and lapatinib-acquired resistance breast cancer cell lines. Upon FTY720 treatment, PP2A was activated and reduced the phosphorylation level of eEF2, while this phenomenon was reversed by the PP2A inhibitor, okadaic acid. Therefore, PP2A and phosphorylation status of eEF2 contributed to the acquired resistance of trastuzumab and lapatinib targeted therapy in HER2 overexpressed breast cancer [101].

**Fig. 5:**
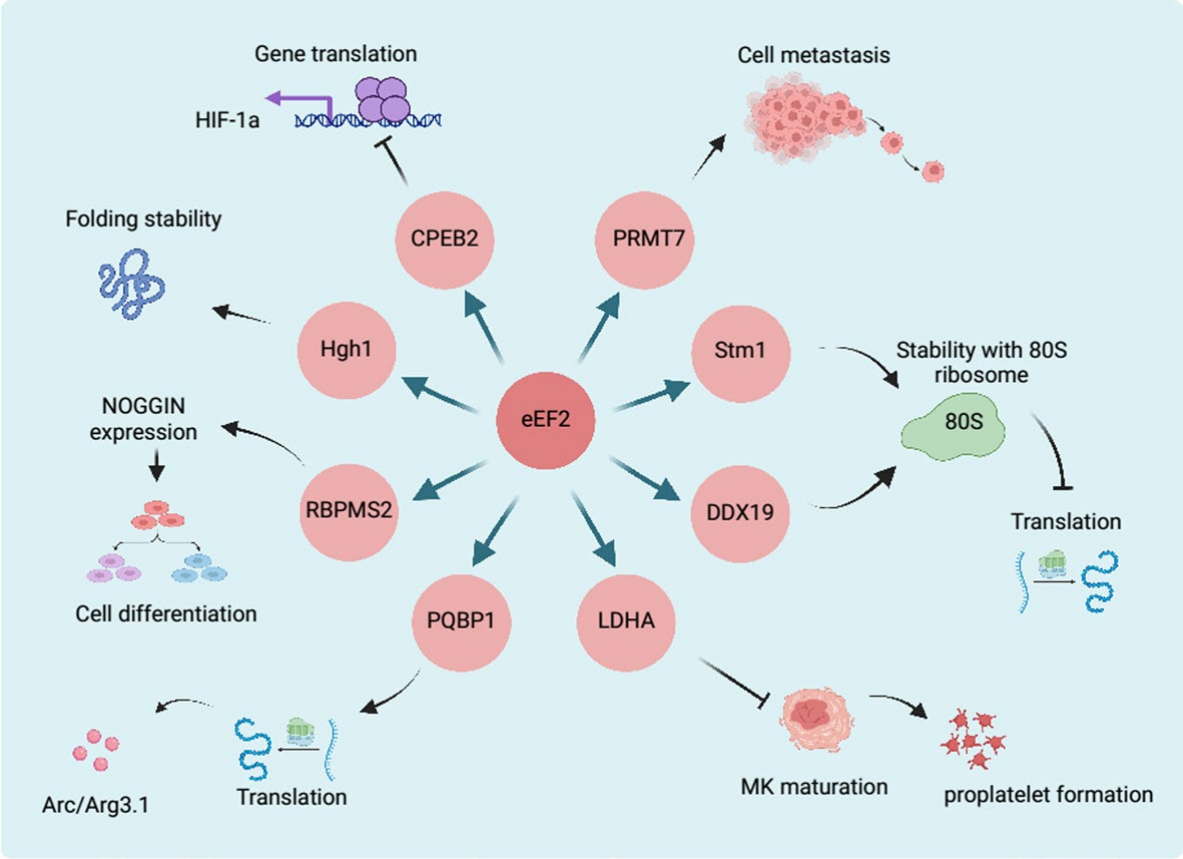
The other reported proteins that contacted with eEF2 and regulated its functions. The figure was created with BioRender.com

In addition to phosphorylation regulators, eEF2 is also regulated by other molecules (Fig. 5). Reports indicated that eEF2 interacted with RBPMS2, causing the accumulation of NOGGIN mRNA and driving the dedifferentiation of smooth muscle cells [102]. Hgh1, an armadillo repeat protein which is afforded to bind to the domain III of eEF2, has been reported to regulate the folding and stability of eEF2, thereby preventing its unproductive interactions and ensuring the N-terminal GTPase is accurately folded [20, 103]. Cytoplasmic polyadenylation element binding protein 2 (CPEB2) was confirmed to reduce the translation of HIF1ɑ via interacting with eEF2 and slowing down its GTP hydrolysis under normoxic environments in HeLa cell lines [104]. In non-small-cell lung cancer, the overexpressed protein arginine methyltransferase 7 (PRMT7) could interact with eEF2, thus promoting the cell metastasis [18]. The stress-related protein Stm1 was found to bind with eEF2 and enhance its stability on the 80S ribosome, therefore suppressing the translation process [105, 106]. Moreover, RNA helicase DDX19 affects protein elongation via stabilizing the complex with eEF2 and ribosome [107]. Lactate dehydrogenase A (LDHA), a glycolysis enzyme which responds for converting pyruvate to lactate, is found to directly interact with eEF2 in megakaryocyte (MK) maturation process [108]. In the presence of nicotinamide adenine dinucleotide (NADH), LDHA recruited eEF2 at its binding pool in cell cytoplasm and down regulated the protein translation process, thereby decreasing the megakaryocyte maturation and thrombocytopoiesis. Recently, polyglutamine binding protein 1 (PQBP1) has been found to interact with eEF2 to regulate the metabolism of the metabotropic glutamate receptor-dependent long-term depression (mGluR-LTD) in recent years [8]. In the cytoplasm, PQBP1 directly interacted with non-phosphorylated eEF2 beside Thr56 amino acid and suppressed the eEF2k dependent phosphorylation of eEF2, thus promoting the protein synthesis and regulating mGluR-LTD.

## Potential inhibitors targeting eEF2 and its associated complexes

**Fig. 6:**
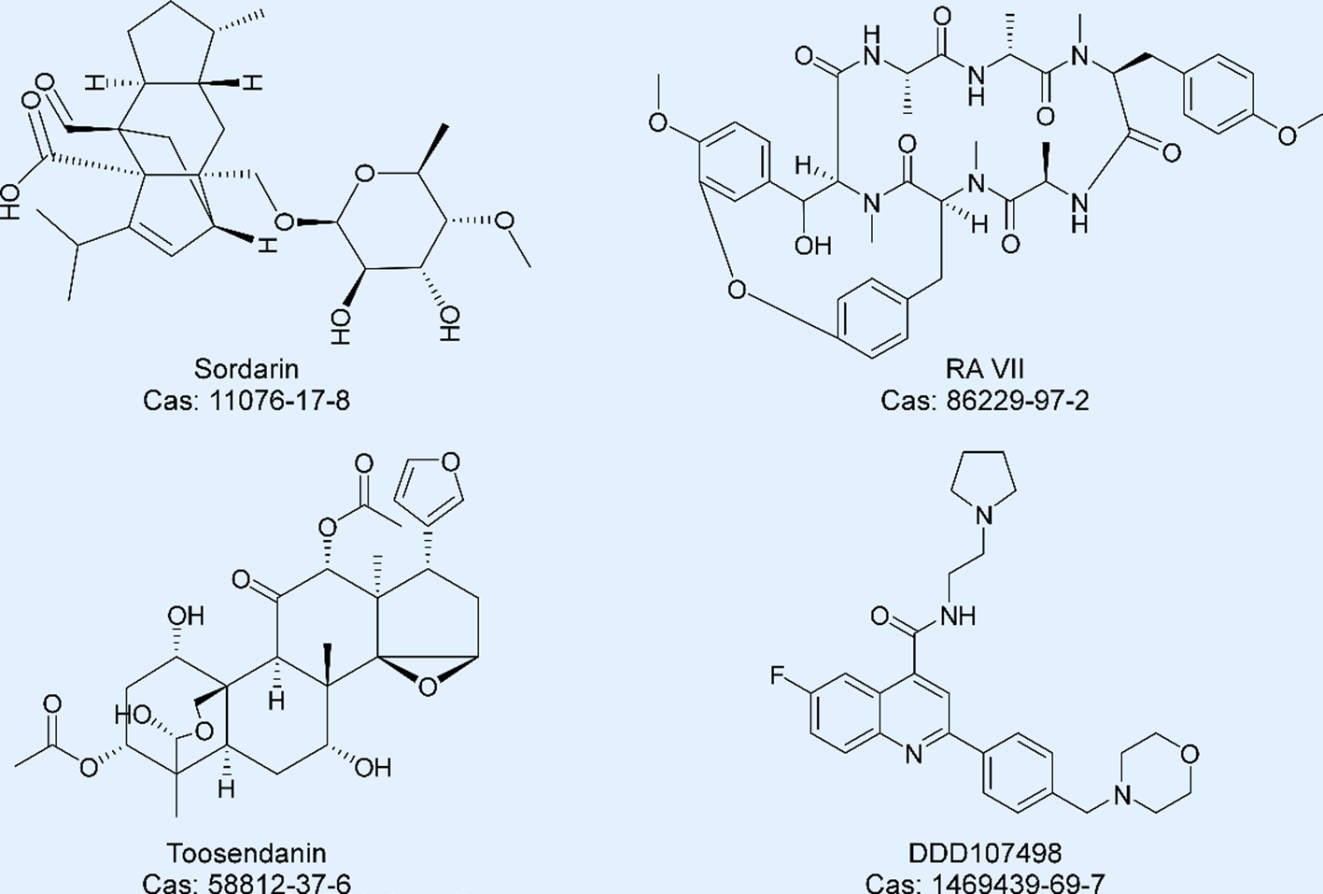
The chemical structure and CAS number of Sordarin, RA-VII, Toosendanin, and DDD107498. The figure was created with ACDSee Systems Canvas

Although numerous compounds have been discovered to inhibit eEF2-related molecular signaling and enhance its phosphorylation level in different cancer cells, there is a scarcity of reported inhibitors that directly target eEF2 or its associated complexes [64, 109, 110]. Currently, the structures of potential inhibitors targeting eEF2 or its associated complexes, including sordarin, RA-VII, toosendanin, and DDD107498, are shown in Fig. 6.

Sordarin is derived from different fungal species and exhibits antifungal effects [111]. Previous studies have indicated that sordarin binds with eEF2 between the III, IV, and V domains [112], specifically interacting with the amino acids in the region of eEF2 from 510 to 567 [113]. This region was accurately narrowed down to the 518–524 amino acids, which is identified as a sordarin-specific region (SSR) and is different from fungal and mammalian eEF2 [114]. Owing to the difference in hydrophilicity of SSR between yeast and human, the binding cavity of eEF2 is altered drastically, leading to sordarin being unable to bind with human eEF2 [115–117]. Sordarin is reported to selectively bind with the GDP-eEF2-ribosome complex and increase its stability and half-life time [118, 119]. Different molecular docking models and cryo-electron microscopy, such as the sordarin-eEF2 and sordarin-eEF2-ribosome complex models, are used to obtain the binding affinity between sordarin and eEF2 [36, 44, 117, 120]. Nevertheless, it has been reported that sordarin exhibits no inhibitory effects on the GTPase activity of eEF2 [121].

RA-VII is a natural cyclic hexapeptide that has been previously isolated from Rubia cordifolia and reported to exhibit anticancer effects [122, 123]. Recently, Miyoshi et al. proposed that the underlying mechanism of RA-VII involves the suppression of protein translation process. In their report, RA-VII selectively targeted eEF2/ribosome rather than eEF-1A/ribosome to inhibit polyphenylalanine synthesis in vitro. As previously described, the binding and dissociation between eEF2 and GTP occur sequentially during translation [6]. Miyoshi et al. found that RA-VII strengthened the binding ability between eEF2 and GTP while reducing GTP exchange, ultimately disrupting eEF2 dissociation from the ribosome after the translocation stage. However, detailed binding sites between RA-VII and eEF2 remain undiscovered; further research is required to confirm the precise regulation mechanism.

Toosendanin (TSN) is a natural compound that is derived from Melia toosendan and has been used as an antiparasitic medicine in China [124]. In recent years, TSN has shown anticancer effects and can suppress the proliferation, migration, and invasion of pancreatic cancer cells, and it suppressed pancreatic cancer progression via down regulating Akt/mTOR signaling [125]. TSN also induced AGS and HGC-27 human gastric cancer cell apoptosis through the p38 MAPK pathway [126]. Recently, our group found TSN could suppress esophageal cancer growth in vitro and in vivo [127]. In the study, eEF2 was highly expressed in esophageal cancer and knocking down of eEF2 suppress tumor cell proliferation and colony formation. We verified that TSN could selectively bind with eEF2 and inhibits its GTPase activity. Additionally, we found that TSN impedes the growth of esophageal cancer via targeting eEF2 and downregulating the protein synthesis of topoisomerase I and II.

In a previous study, DDD107498 was developed as a novel antimalarial agent with good pharmacokinetic features [128, 129]. It exhibited antimalarial effects by blocking various life-cycle stages of the parasite and was modified on the basis of the 2,6-disubstituted quinoline-4-carboxamide compound screened from a vast compound series. Toxicity studies indicated minimal effects on human cytochrome P450, suggesting that it is safe to combine with other drugs. Whole genome sequencing and single nucleotide polymorphism verification confirmed eEF2 as the target of DDD107498. However, further research is necessary to assess how DDD107498 interacts with eEF2 and its inhibitory effects on cancer. Developing new inhibitors targeting eEF2 remains urgent for both basic and clinical study purposes.

### Strategies to inhibit the function of eEF2 in cancer

Targeting eEF2 GTPase activity: since eEF2’s GTPase activity is crucial for its role in translation elongation, inhibitors, such as TSN, that target GTP hydrolysis offer promising therapeutic potential [127]. Developing small molecules to specifically bind and inhibit the GTP-binding pocket of eEF2 could directly block translation elongation, thus inhibiting cancer cell growth. Targeting post-translational modifications (PTMs) of eEF2: eEF2 is regulated by PTMs, such as phosphorylation (via eEF2K) [13, 62] and methylation (via FAM86A) [59]. Targeting these modifications could disrupt eEF2’s function in translation. For instance, inhibitors that prevent eEF2 phosphorylation or methylation could lock eEF2 in its inactive form, thereby reducing its role in tumor proliferation. Blocking protein complex formation: disrupting interactions between eEF2 and other translation regulatory proteins, such as the ribosome, or elongation factors, such as eEF1A, may also serve as an effective strategy. Molecules, such as RA-VII, show promise in this area, although further studies are necessary to elucidate their precise mechanisms [122]. Identifying specific inhibitors that block these protein complexes could help fine-tune this therapeutic approach.

In conclusion, integrating these strategies targeting eEF2’s GTPase activity, modulating PTMs, and blocking complex formation-offers a comprehensive framework for inhibiting eEF2 in cancer therapy. Further research is needed to refine these strategies for clinical application.

## Conclusions

**Fig. 7:**
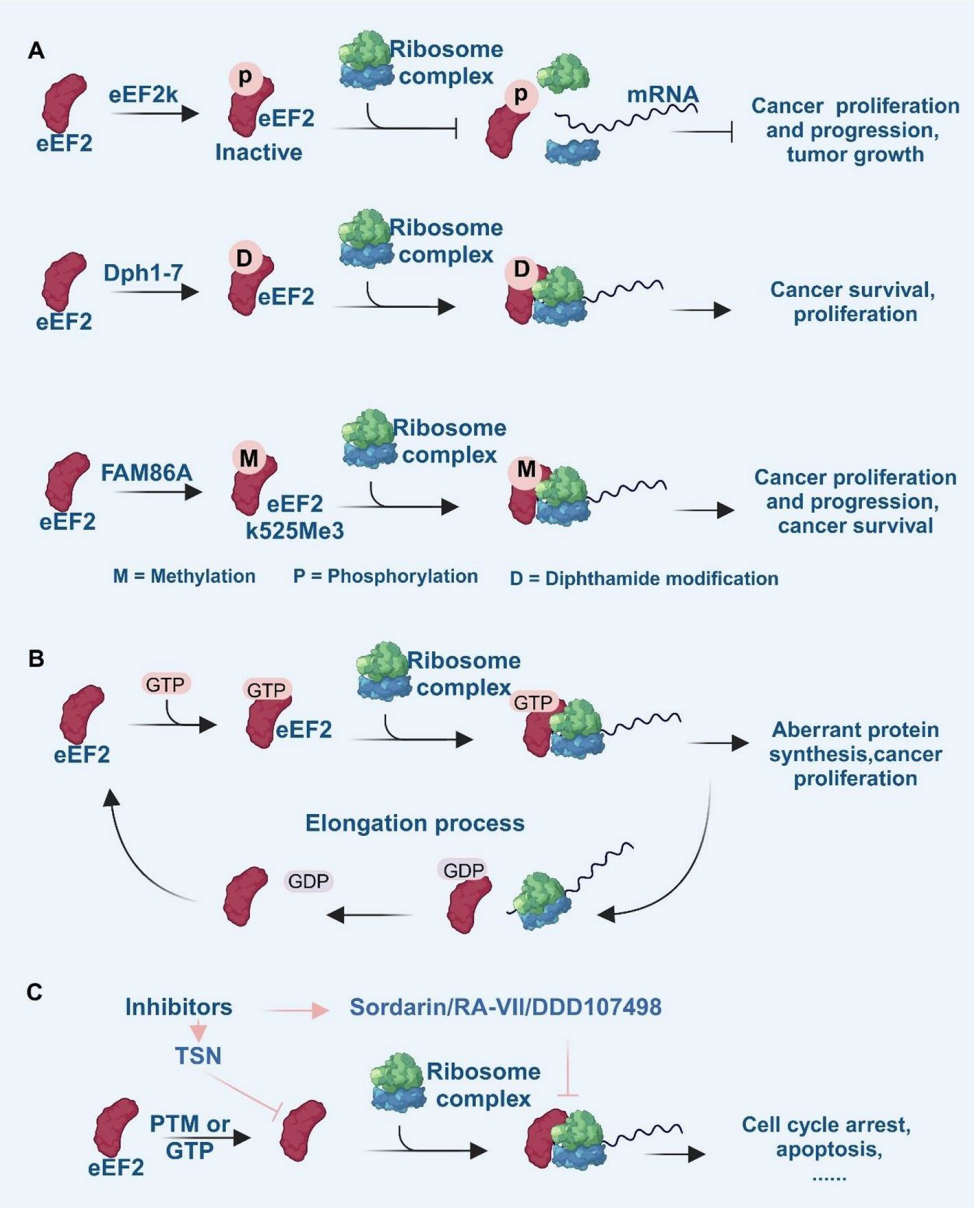
Illustration of eEF2 targeting strategies in cancer therapy: modulation of posttranslational modification (PTM), GTPase activity, and inhibitor strategies. **A** Posttranslational modifications of eEF2: phosphorylation of eEF2 by eEF2K renders it inactive, suppressing its integration into the elongation complex and thereby inhibiting cancer cell proliferation and progression. In contrast, diphthamide modification on eEF2 facilitates its binding to mRNA and stabilizes its interaction with the ribosome complex, promoting cancer cell survival and proliferation. Similarly, methylation of eEF2, catalyzed by FAM86A, enhances eEF2’s interaction with the ribosome, further driving cancer progression. **B** GTPase activity of eEF2: eEF2 binds to GTP, facilitating the formation of the ribosome–protein synthesis complex. Following GTP hydrolysis to GDP, eEF2 dissociates from the ribosome, initiating the next elongation cycle. Aberrant activation of this process results in abnormal protein synthesis, leading to enhanced cancer cell proliferation. **C** Therapeutic targeting of eEF2 and its complexes: targeting eEF2 or its associated complexes can suppress cancer proliferation by disrupting translation processes, inducing cell cycle arrest, and triggering apoptosis. The figure was created with BioRender.com

In summary, eEF2 plays multiple roles in the elongation process and affects translation steps in both normal and cancer cells. Aberrant eEF2 expression and dysregulated signaling can induce abnormal protein synthesis in cancer cells, thereby promoting cell proliferation. In this review, we summarize the translation process involving eEF2 as follows Fig. 7: during translation, phosphorylation or dephosphorylation of eEF2 regulates the activation switch during protein synthesis regulation, with its activation status identifying as an indicator for elongation. Diphthamide modification on eEF2 enables its binding to mRNA and facilitates its function with the ribosome complex, thereby promoting protein synthesis. Additionally, methylation of eEF2 catalyzed by FAM86A, enhances eEF2’s interaction with the ribosome, promoting protein synthesis and cancer cell proliferation.

As a member of the GTPase family, eEF2 binds to GTP and promotes the formation of ribosome-protein synthesis complex. Following GTP hydrolysis to GDP by eEF2, the GDP-eEF2 complex dissociates from the ribosome complex, initiating the next elongation cycle [6]. However, further exploration is required regarding eEF2’s contribution to GTP hydrolysis. Additionally, investigating transient translocation functions of eEF2 may provide insights into its detailed mechanism since crystal structure models cannot capture its dynamic processes accurately.

In inhibitor research, the text underscores the significance of understanding the intricate mechanisms of eEF2 in protein translation and their implications in cancer progression. Targeting eEF2 with inhibitors presents an innovative avenue for cancer therapy; however, more research is needed to unravel the exact binding sites and regulatory mechanisms these inhibitors have on eEF2. As advancements continue in this field, the development of novel eEF2 inhibitors holds great promise for enhancing the efficacy of cancer treatments by disrupting critical pathways that support cancer cell growth and survival. The pursuit of new eEF2 inhibitors not only contributes to fundamental scientific knowledge but also holds the potential to translate into valuable clinical applications.

Additionally, future investigations should also explore how eEF2’s phosphorylation status affects its structure and elongation process. The cross-link between eEF2 GTPase, phosphorylation, and SUMOylation in the translation process remains incompletely understood. Moreover, despite being highly expressed in various cancers, the posttranslational modifications of eEF2 have not been thoroughly studied. Notably, recent findings suggest that the phosphorylation status of eEF2 differs between in vivo and in vitro cancer cells exposed to compound treatments; thus, elucidating the underlying mechanisms and identifying sensitive inhibitors is crucial for advancing both basic and clinical research.

## Supplementary Information

Supplementary Material 1.

Supplementary Material 2.

## Data Availability

Not applicable.

## Abbreviations

eEF2: Eukaryotic elongation factor 2
TORC1: The classical rapamycin complex1
NH125: 1-Benzyl-3-cetyl-2-methylimidazolium iodide
CPEB2: Cytoplasmic polyadenylation element binding protein 2
PRMT7: Protein arginine methyltransferase 7
LDHA: Lactate dehydrogenase A
PQBP1: Polyglutamine binding protein 1
MK: Megakaryocyte
TSN: Toosendanin
