# Supplementary material for: Elongation factor 2 in cancer: a promising therapeutic target in protein translation
Source: Cell Mol Biol Lett. 2024 Dec 20;29:156. doi: 10.1186/s11658-024-00674-7 (PMC11660736; doi:10.1186/s11658-024-00674-7)
Supplement: Supplementary file 2 — Supplementary Material 2. [file 11658_2024_674_MOESM2_ESM.pdf]

```
                         1        10        20        30        40        50
sp|P13639|EF2_HUMAN      MVNFTVDQIRAIMDKKANIRNMSVIAHVDHGKSTLTDSLVCKAGIIASARAG..ETRF
sp|P13060|EF2_DROME      MVNFTVDEIRGLMDKKRNIRNMSVIAHVDHGKSTLTDSLVSKAGIIAGAKAG..ETRF
sp|P32324|EF2_YEAST      MVAFTVDQMRSLMDKVTNVRNMSVIAHVDHGKSTLTDSLVQRAGIISAAKAG..EARF
sp|P0A6M8|EFG_ECOLI      .........MARTTPIARYRNIGISAHIDAGKTTTTERILFYTGVNHKIGEVHDGAAT

                           60        70        80        90        100       110
sp|P13639|EF2_HUMAN      TDTRKDEQERCITIKSTAISLFYELSENDLNFIKQ....SKDGAGFLINLIDSPGHVD
sp|P13060|EF2_DROME      TDTRKDEQERCITIKSTAISMYFEVEEKDLVFITHPDQREKECKGFLINLIDSPGHVD
sp|P32324|EF2_YEAST      TDTRKDEQERGITIKSTAISLYSEMSDEDVKEIKQ....KTDGNSFLINLIDSPGHVD
sp|P0A6M8|EFG_ECOLI      MDWMEQEQERGITITSAATTAFWSG.............MAKQYEPHRINIIDTPGHVD

                                 120       130       140       150       160
sp|P13639|EF2_HUMAN      FSSEVTAALRVTDGALVVVDCVSGVCVQTETVLRQAIAERIKPVLMMNKMDRALLELQ
sp|P13060|EF2_DROME      FSSEVTAALRVTDGALVVVDCVSGVCVQTETVLRQAIAERIKPILFMNKMDRALLELQ
sp|P32324|EF2_YEAST      FSSEVTAALRVTDGALVVVDTIEGVCVQTETVLRQALGERIKPVVVINKVDRALLELQ
sp|P0A6M8|EFG_ECOLI      FTIEVERSMRVLDGAVMVYCAVGGVQPQSETVWRQANKYKVPRIAFVNKMDRMGANFL

                        170       180       190       200       210       220
sp|P13639|EF2_HUMAN      LEPEELYQTFQRIVENVNVIISTYGEGESGPMGNIMIDPVLGTVGFGSGLHGWAFTLK
sp|P13060|EF2_DROME      LDAEELYQTFQRIVENVNVIIATYND.DGGPMGEVRVDPSKGSVGFGSGLHGWAFTLK
sp|P32324|EF2_YEAST      VSKEDLYQTFARTVESVNVIVSTYAD...EVLGDVQVYPARGTVAFGSGLHGWAFTIR
sp|P0A6M8|EFG_ECOLI      KVVNQIKTRLG.........................ANPVPLQLAIGAEEHFTGVVD.

                           230       240       250       260       270       280
sp|P13639|EF2_HUMAN      QFAEMYVAKFAAKGEGQLGPAERAKKVEDMMKKLWGDRYFDPANGKFSKSATSPEGKK
sp|P13060|EF2_DROME      QFSEMYSEKFKID.............VVKLMNRLWGENFFNAKTKKWQKQKEADN...
sp|P32324|EF2_YEAST      QFATRYAKKFGVD.............KAKMMDRLWGDSFFNPKTKKWTNKDTDAEGKP
sp|P0A6M8|EFG_ECOLI      ..........................LVKMKAINWNDADQGVTFEYEDIP........

                             290       300       310       320       330       340
sp|P13639|EF2_HUMAN      LPRTFCQLILDPIFKVFDAIMNFKKEETAKLIEKLDIKLDSEDKDKEGKPLLKAVMRR
sp|P13060|EF2_DROME      .KRSFCMYILDPIYKVFDAIMNYKKEEIGTLLEKIGVTLKHEDKDKDGKALLKTVMRT
sp|P32324|EF2_YEAST      LERAFNMFILDPIFRLFTAIMNFKKDEIPVLLEKLEIVLKGDEKDLEGKALLKVVMRK
sp|P0A6M8|EFG_ECOLI      ......ADMVELANEWHQNLIESAAEASEELMEKYLGGEELTEAEIKG.....ALRQR

                               350       360       370       380       390       400
sp|P13639|EF2_HUMAN      WLPAGDALLQMITIHLPSPVTAQKYRCELLYEGPPDDEAAMGIKSCDPKGPLMMYISK
sp|P13060|EF2_DROME      WLPAGEALLQMIAIHLPSPVVAQKYRMEMLYEGPHDDEAAIAVKSCDPDGPLMMYISK
sp|P32324|EF2_YEAST      FLPAADALLEMIVLHLPSPVTAQAYRAEQLYEGPADDANCIAIKNCDPKADLMLYVSK
sp|P0A6M8|EFG_ECOLI      VLNNEIILVTCGSAFKNKGVQAMLDAVIDYLPSPVDVPAINGILDDGKDTPAERHASD

                                 410       420       430       440       450
sp|P13639|EF2_HUMAN      MVPTSDKGRFYAFGRVFSGLVSTGLKVRIMGPNYTPGKKEDLYLKPIQRTILMMGRYV
sp|P13060|EF2_DROME      MVPTSDKGRFYAFGRVFAGKVATGQKCRIMGPNYTPGKKEDLYEKAIQRTILMMGRYV
sp|P32324|EF2_YEAST      MVPTSDKGRFYAFGRVFAGTVKSGQKVRIQGPNYVPGKKDDLFIKAIQRVVLMMGRFV
sp|P0A6M8|EFG_ECOLI      DEPFSALAFKIATDPFVGNLTFFRVYSGVVNSGDTVLNSVKAARERFGRIVQMHANKR

                        460       470       480       490       500       510
sp|P13639|EF2_HUMAN      EPIEDVPCGNIVGLVGVDQFLVKTGTITTFEHAHNMRVMKFSVSPVVRVAVEAKNPAD
sp|P13060|EF2_DROME      EAIEDVPSGNICGLVGVDQFLVKTGTITTFKDAHNMKVMKFSVSPVVRVAVEPKNPAD
sp|P32324|EF2_YEAST      EPIDDCPAGNIIGLVGIDQFLLKTGTLTTSETAHNMKVMKFSVSPVVQVAVEVKNAND
sp|P0A6M8|EFG_ECOLI      EEIKEVRAGDIAAAIGLK..DVTTGDTLCDPDAPIILERMEFPEPVISIAVEPKTKAD

                           520       530       540       550       560       570
sp|P13639|EF2_HUMAN      LPKLVEGLKRLAKSDPMVQCIIEE.SGEHIIAGAGELHLEICLKDLEEDHACIPIKKS
sp|P13060|EF2_DROME      LPKLVEGLKRLAKSDPMVQCIIEE.SGEHIIAGAGELHLEICLKDLEEDHACIPLKKS
sp|P32324|EF2_YEAST      LPKLVEGLKRLSKSDPCVLTYMSE.SGEHIVAGTGELHLEICLQDLEHDHAGVPLKIS
sp|P0A6M8|EFG_ECOLI      QEKMGLALGRLAKEDPSFRVWTDEESNQTIIAGMGELHLDIIVDRMKREFN.VEANVG

                              580       590       600       610       620       630
sp|P13639|EF2_HUMAN      DPVVSYRETVSEESNVLCLSKSPNKHNRLYMKARPFPDGLAEDIDKGEVSARQELKQR
sp|P13060|EF2_DROME      DPVVSYRETVSEESDQMCLSKSPNKHNRLLMKALPMPDGLPEDIDNGDVSAKDEFKAR
sp|P32324|EF2_YEAST      PPVVAYRETVESESSQTALSKSPNKHNRIYLKAEPIDEEVSLAIENGIINPRDDFKAR
sp|P0A6M8|EFG_ECOLI      KPQVAYRETIRQKVT...........................DVEGKHAKQSGGRGQY

                                640       650       660       670       680
sp|P13639|EF2_HUMAN      ARYLAEKYEWDVAEARKIWCFGPDGTGPNILTDITKGVQYLNEIKDSVVAGFQWATKE
sp|P13060|EF2_DROME      ARYLSEKYDYDVTEARKIWCFGPDGTGPNFILDCTKSVQYLNEIKDSVVAGFQWASKE
sp|P32324|EF2_YEAST      ARIMADDYGWDVTDARKIWCFGPDGNGPNLVIDQTKAVQYLHEIKDSVVAAFQWATKE
sp|P0A6M8|EFG_ECOLI      GHVVIDMYPLEPGSNPKGYEFINDIKGG..........VIPGEYIPAVDKGIQEQLKA
```

```
                    690       700       710       720       730       740
sp|P13639|EF2_HUMAN GALCEENMRGVRFDVHDVTLHADAIHRGGGQIIPTARRCLYASVLTAQPRLMEPIYLV
sp|P13060|EF2_DROME GILADENLRGVRFNIYDVTLHADAIHRGGGQIIPTTRRCLYAAAITAKPRLMEPVYLC
sp|P32324|EF2_YEAST GPIFGEEMRSVRVNILDVTLHADAIHRGGGQIIPTMRRATYAGFLLADPKIQEPVFLV
sp|P0A6M8|EFG_ECOLI GPLAGYPVVDMGIRLHFGSYHDVDSSE..LAFKLAASIAFKEGFKKAKPVLLEPIMKV

                      750       760       770       780       790       800
sp|P13639|EF2_HUMAN EIQCPEQVVGGIYGVLNRKRGHVFEESQVAGTPMFVVKAYLPVNESFGFTADLRSNTG
sp|P13060|EF2_DROME EIQCPEVAVGGIYGVLNRRRGHVFEENQVVGTPMFVVKAYLPVNESFGFTADLRSNTG
sp|P32324|EF2_YEAST EIQCPEQAVGGIYSVLNKKRGQVVSEEQRPGTPLFTVKAYLPVNESFGFTGELRQATG
sp|P0A6M8|EFG_ECOLI EVETPEENTGDVIGDLSRRRGMLK..GQESEVTGVKIHAEVPLSEMFGYATQLRSLTK

                        810       820       830       840       850
sp|P13639|EF2_HUMAN GQAFPQCVFDHWQILPGDPFDNSSRPSQVVAETRKRKGLKEGIPALDNFLDKL
sp|P13060|EF2_DROME GQAFPQCVFDHWQVLPGDPSEPSSKPYAIVQDTRKRKGLKEGLPDLSQYLDKL
sp|P32324|EF2_YEAST GQAFPQMVFDHWSTLGSDPLDPTSKAGEIVLAARKRHGMKEEVPGWQEYYDKL
sp|P0A6M8|EFG_ECOLI GRASYTMEFLKYDEAPSN.......VAQAVIEARGK.................
```
